# Some Observations on Conception and Pregnancy in the Mare

**Published:** 1897-04

**Authors:** J. A. Dell

**Affiliations:** Ann Arbor, Michigan


### SOME OBSERVATIONS ON CONCEPTION AND PREGNANCY IN THE MARE.

BY J. A. DELL, V.S.,

ANN ARBOR, MICHIGAN.

(Concluded from page 124.)

WHILE the cessation of the periods of heat must be accepted as well-founded proof of conception, still there are cases reported where they have been said to reappear more or less regularly and still the mare be pregnant. In proof of the latter I have failed to establish anything definite. It is true that many mares have been bred to stallions in the usual way, yet given birth to foals from former service; but it is very doubtful, if allowed their freedom, that they would copulate. I have never seen nor can I find any record where mares running with a stallion have ever copulated when pregnant.

I now own a little Shetland mare, seven months pregnant, that, each day when turned into the paddock with other mares and the stallion, regularly goes toward the stallion, neighs, and shows what would ordinarily be taken as signs of heat, yet she will not receive him.

It is said that the bull, when allowed to run with the herd, refuses to give any attention to the pregnant cows, and I believe it is so with the stallion, if allowed to range with mares continuously. Even some stallions kept away as usual seem to know by instinct and refuse to serve a mare in foal.

An instance of that kind occurred in our city a few years ago. A mare supposed to be in heat was brought to be bred. When brought near the stallion she evinced marked signs of heat, yet he refused to serve her, and the owner took her away, disgusted with the stallion, yet in less than two months she gave birth to a strong, healthy foal. But I doubt if that mare had been turned with a stallion used to ranging with mares if she would have copulated.

I am of the opinion that the so-called heat is rarely much more than nervousness. With mares well broken, as most of our horses now are, and the usual amount of force, they submit to service when they would not if allowed their liberty.

Some writers have attempted to prove this as well as superfœtation by mares giving birth to a horse and a mule foal from covers several days apart; but when we remember that one heat may continue for nearly three weeks, it is safe to assume that both services were during the one heat.

Following the cessation of heat we have the material signs, such as increase in the volume of the abdomen, tendency to fatten, changes in temperament, etc., to all of which I have failed to add anything reliable. In fact, I have failed to establish anything definite from them, as food, attention, and surroundings conduce to the same conditions; and, as I have before stated, it has all but too often been the case in the past decade that a hay colt has resulted from a high-priced service.

With all due respect to our writers and teachers who place great reliance on some of the above symptoms, together with measurements of the abdomen, increase in weight, examination of the urine or examinations per rectum or vagina, or through the abdominal walls for the tumorous condition of the gravid uterus, I believe there is one, and only one, true sign of pregnancy in the mare on which the veterinarian can risk his professional opinion; that is, life in the fœtus, and it devolves upon us to discover how and how soon we can be able to detect that important period.

You are all well aware that, due to the action of the panniculus carnosus, the thickness of the walls, together with the normal sounds in the abdomen, the stethoscope, so valuable an instrument in examining the human patient, is entirely impracticable in the larger veterinary patients; neither are we able to detect anything during the early stages of pregnancy with the naked ear. Thus our observations are narrowed down to the one sensation, that of touch. Fleming gives three modes by which the fœtus may be felt in utero: "By the vagina, the rectum, or through the abdominal walls."

The first I should name as the most unsatisfactory; the straining that follows examinations of the vagina is disagreeable, to say nothing about the danger that may follow in some very sensitive mares; while I have never been able to detect anything more than the distended uterus, except in cases very near parturition, and sometimes not then, as the fœtus had settled low in the abdominal cavity, and nothing but the fluids distended that part.

That by the rectum, while much safer, is likewise very unsatisfactory. Fleming states that after three months the tumor-like gravid uterus can be felt by this means; but when we take his statement that at that time the fœtus of an ordinary sized mare is six inches or less long, that the envelopes are from four to eight times the weight of the fœtus, and that the fluids are from six to ten times its weight—surrounded by all this, covered by the walls of both uterus and rectum—the man who can detect it with certainty has a far more delicate touch than I have been able to cultivate.

If the fœtus exhibited life at that period, we would have but little difficulty in deciding; but when we are informed that the human mother first observes life in her unborn child at about four and one-half months, or midterm, how can one expect to detect it in the mare through so much intervening media at an earlier period? Besides, as we are all aware, as the fœtus grows in size its weight tends to carry it lower in the abdomen, and by the time it has acquired size and strength enough to exhibit much movement it has sunken so low that it is nearer the inferior abdominal wall than the rectum, as all of us have found who have made post-mortems on pregnant mares that were nearly half-gone. When opening the abdomen the first thing met was the gravid uterus, and this position I believe it assumes about the close of the fourth month of pregnancy, displacing all other viscera, and rests directly on the abdominal walls, and for these reasons I have found the rectal examination difficult and very unreliable.

Before entering upon modes of external examination let us first consider the position of the fœtus in utero, that we may have a better idea of what part of the fœtus we expect to meet and at what part of the maternal abdomen we shall be most liable to first feel it. I agree with all those I have consulted that the normal position of the fœtus in the mare is one or the other of the cornu, with the head directed toward the cervix, and that position I believe it maintains during its entire period of development. As proof of it, I never made a post-mortem on any pregnant mare and found it differently; also in premature births that presentation is as constant as in full-term births; and, again, in mummified, immature fœtuses that I have removed I have found them in that position That the fœtus lies on its side or back, with its hind feet extending forward into the cornu of the uterus during its development, and turns itself only at the approach of parturition, as some of our best authors state, I do not believe, for in all post-mortems I have found the fœtus resting on its abdomen with front and hind feet curled under it, and further on I will give other reasons to substantiate my theory; also that the fœtus rests more in a sitting posture until well developed—that is, its posterior extremities rest nearer the abdominal walls of the dam than the anterior.

After discarding both vaginal and rectal examinations I gave more attention to manipulating the abdomen. In this way Fleming claims it is not reliable before the seventh or eighth month, and I am not surprised that he did not detect life earlier if he followed the directions he gives. He says: "place your hand on the abdomen just in front of the mammary glands." Now, when we stop to reason, would that be the point at which the gravid uterus in its descent would be first to touch the abdominal walls? The cornu in which the fœtus develops has its anterior and posterior attachments, and in its normal state it forms the segment of a circle, the most dependent part above or anterior to the umbilicus, and is it not reasonable to suppose that as it enlarges it would settle down from that point, and in that region we must look for the earliest indications of life?

In my earliest examinations, by exercising a little patience, I found no difficulty in detecting life at seven months, the motions at that period being of the nature of a small plunge or jump; I liken them to the actions of a small pig in a sack. Taking notice of the minor motions, and making examinations of all cases I met where the time was known, I soon found at six months I could readily detect life; but the motions at that period are less strong and can be said to be a quiver or flutter, likened to a fowl in a sack.

When I became conversant with the movements of a six-months' fœtus I was led to believe that it was not for lack of movements that we could not detect life earlier, but to our inability to recognize the delicate movements. In 1894 I had mares of my own at my stables constantly, and I examined them two or three times a week, and the earliest I was able to be positive that year was five months and six days. But now I can recall observing movements earlier that I have since proved to be definite signs.

The past season I have had similar opportunities, and I began my examinations as early as three months, but was not positive with the first until the even five months; but in the second I detected movements at four months and twenty-six days, while later I was positive of movements in one at four months and nineteen days and another at four months and eighteen days. What was very interesting to me was, after making these early detections, to examine them from week to week thereafter and note the increase and strength of movements as the period increased.

The movements at four months and eighteen days vary as much from movements at five months as those from six to seven months, or seven to eight months, etc.; and, while we may never be able to detect positive life earlier than this minimum period, I do believe that with frequent opportunity for examination and the cultivation of our sense of touch we may become so well acquainted with the different movements as to be able not only to detect pregnancy, but to give a reasonable opinion as to what stage it has reached.

When making an examination I stand at the side of the mare. I prefer the left, as I seem to have a more correct touch with my right hand. I place my hand in the median line, at or just in front of the umbilicus. The very earliest movements I have felt nearly as far forward of the umbilicus as it is forward of the mammary glands.

The earliest movements I liken to the sensation we would receive by covering the palm of the hand with several folds of cloth, then touching it with the tips of the fingers of the other hand, one or two at a time, suddenly changing to the other tips, so as to give a sensation of little thumps or steps, or liken it to the feet of a cat stepping on your bed-cover, only more sudden changes. These movements, I believe, are made with the hind feet or hocks, and convince me, as I stated before, that the fœtus rests in a sitting posture and that the posterior extremities settle and reach the floor of the abdomen first.

At about five months the movements are felt further back and seem more complicated—that is, more touches as with finger-tips—and stronger, and with them we detect a slight quiver. The former, I believe, can be attributed to the enlarging and settling of the fœtus, and we get some of the movements of the fore-feet, while the quivering I believe to be the beginning of the body movements.

At six months these increase in strength, and at about that time we can detect a slight jumping sensation. The latter increases quite rapidly until, by the beginning of the seventh month, the fœtus acquires so much strength that there is little difficulty in detecting it by placing the hand on the lower part of the abdomen. During the eighth month, and sometimes earlier, by close watching we can see movements at the flanks, and these increase in force until, as many of us have observed, they seem to shake the whole body of the dam.

I believe life can be discerned a little sooner in mares that grow large early, as the muscles of the abdomen are then thinner than those that have smaller and harder abdomens. As the best time of day to examine, I have failed to discern much difference; the most of mine have been in the evening after feeding and watering. I have had my best success if I could catch them lying and examine them just as they got on their feet. After drinking of cold water or eating of food, particularly if of a nature to generate much gas, I find a good time. One condition I have observed—that is, if I find marked peristalsis I do not have to wait long to get movements if the fœtus be sufficiently developed.

